# Prevalence of asymptomatic malaria and associated factors among pregnant women in Ethiopia: systematic review and meta-analysis

**DOI:** 10.3389/frph.2023.1258952

**Published:** 2023-10-11

**Authors:** Tadesse Duguma, Eyob Tekalign, Samuel Sahile Kebede, Getachew Mesfin Bambo

**Affiliations:** Department of Medical Laboratory Science, College of Health Science and Medicine, Mizan-Tepi University, Mizan-Aman, Ethiopia

**Keywords:** asymptomatic malaria, prevalence, associated factors, pregnant women, Ethiopia

## Abstract

The proactive identification of asymptomatic patients and the mitigation of associated problems are essential to the elimination of malaria. For asymptomatic malaria and related variables among pregnant women in Ethiopia, there are no national pooled estimates. As a result, the goal of this study is to compile thorough and compelling data from several Ethiopian investigations. Google Scholar, PubMed, Scopes, the Web of Science, the Cochrane Library, and African Journals Online were a few of the electronic resources that were accessed. The investigation included all observational studies. STATA version 15 was used to extract the data from the Microsoft Excel file and conduct the analysis. The estimated pooled prevalence of asymptomatic malaria among pregnant women was calculated using a random-effects model. An inverse variance index (I^2^) analysis was utilized to find heterogeneity. To assess the publication bias, funnel plots, and Egger's statistical tests were used. The study determined that the combined prevalence of asymptomatic malaria among pregnant women was 7.20 (95% confidence interval = 4.22, 10.18) and 4.69 (95% confidence interval = 2.77, 6.62) by microscopy and rapid diagnostic test, respectively. The presence of stagnant water near their home (odds ratio = 4.31; 95% confidence interval = 1.66, 11.20); not using insecticide-treated nets (odds ratio = 6.93; 95% confidence interval = 3.27, 14.71); the lack of indoor residual spray service (odds ratio = 2.68; 95% confidence interval = 1.63, 4.40); and the presence of pregnant women in their neighborhood (odds ratio = 3.14; 95% confidence interval = 1.4). This study showed that pregnant women have a high pooled prevalence of asymptomatic malaria. Women living in rural areas near stagnant water and those who never used insecticide-treated nets had a two-, four-, or six-fold higher prevalence of asymptomatic malaria, respectively. The use of advanced diagnostic techniques could produce a higher magnitude of the disease. For effective intervention toward elimination, active case detection at the community level is also advised.

**Systematic Review Registration:**

https://www.crd.york.ac.uk/prospero/display_record.php?ID=CRD42023411385; identifier, CRD42023411385.

## Introduction

Malaria continues to be a public health issue even though enormous efforts have been made to date for both prevention and treatment. The rates of sickness and mortality from malaria remain intolerably high, decades after the global drive to eradicate it began. The long-term objective of eliminating malaria should be maintained while the international community continues to concentrate on reducing sickness and fatalities. Roll Back Malaria was a great success in reducing morbidity and mortality, even though malaria cases are still alarming in nations with few resources (1, 2).

In 2021, there were 619,000 malaria-related deaths globally, down from 625,000 in the pandemic's first year, according to the World Health Organization (WHO). In 2019, before the pandemic, there had been 568, 000 fatalities. Although they increased more slowly than they did between 2019 and 2020, malaria cases increased between 2020 and 2021. Malaria affected 247 million people globally in 2021, up from 232 million in 2019 and 245 million in 2020. In the WHO African Region, the number of malaria-related deaths declined from 841,000 in 2000 to 541,000 in 2018, then rose to 599,000 in 2020. The predicted death toll dropped once again, to 593,000 in 2021. Between 2000 and 2019, the fatality rate from malaria dropped by 62%, from 148 to 56 per 100,000 individuals at risk, before increasing to 60 in 2020 and then dropping to 58 in 2021 (3).

In the WHO African Region in 2021, 40 million pregnancies are anticipated, 13.3 million (or 32%) of which will be malaria-infected. These pregnancies occurred in 38 countries with medium-to-high malaria transmission rates. In terms of exposure to malaria during pregnancy, the WHO subregion of West Africa had the greatest prevalence (40.7%), closely followed by Central Africa (39.8%), while the prevalence in East and Southern Africa was 20%. Global and African data show that there were 204 million fewer malaria cases in Africa in 2019 than there were in 2000 and that the incidence of malaria cases reduced during this time from 363 to 225 cases per 1,000 people at risk (3, 4).

The incidence and death of malaria have been significantly decreased by interventions that target both the parasites and the vector, such as artemisinin-based combination therapy (ACT), insecticide-treated nets (ITNs), and indoor residual spraying (IRS). As a result of this achievement in reducing malaria incidence and mortality, there is currently low to moderate malaria transmission in several nations (5).

Asymptomatic malaria poses a significant challenge to the elimination of malaria in all countries where it is endemic. Information on asymptomatic malaria is essential to enhance estimates of malaria prevalence and support malaria elimination efforts. In endemic areas, due to innate immunity, asymptomatic malaria parasitemia—the presence of asexual parasites in the blood without symptoms of illness—can occur, which is brought on by genetic factors, malaria infection leading to low parasite density, and naturally occurring immunity to the disease (6–11). Asymptomatic malaria infection may lead to a reduced gametocyte density, which can nonetheless cause malaria transmission (8, 12). Understanding malaria epidemiology is crucial for the elimination of the disease, as it is more effective to stop the transmission from asymptomatic individuals than to deliver large doses of medication during low-transmission conditions (12, 13). Asymptomatic malaria's prevalence varies by demographic type, transmission environment, study design, and technique of diagnosis, according to research done so far (10, 14, 15).

Due to the sharp decline in malaria incidence and mortality as well as the rapidly evolving malaria risk map, Ethiopia has set a goal to eradicate malaria nationwide by 2030 (16, 17). To achieve elimination, it is crucial to comprehend the local malaria condition, especially the incidence of asymptomatic malaria and associated risk factors. Despite only a few small-scale studies being conducted nationwide, the objective of this systematic review and meta-analysis was to determine the pooled prevalence of asymptomatic malaria and its related factors at the national level.

Estimates will be more precise if they consider any possible heterogeneity as well as the aggregate prevalence of asymptomatic malaria infection and associated variables among pregnant women. We may also learn in-depth information about a variety of elements of asymptomatic malaria that we cannot learn through individual research. Understanding the incidence of asymptomatic malaria in various regions of the nation and the various risk variables to consider for targeted therapy is particularly crucial. The results of this study can also be used to develop national strategies that take the geographical effects of asymptomatic malaria into account and to better understand how common asymptomatic malaria is in pregnant Ethiopian women. In addition, this study will point out any gaps in the body of knowledge regarding the difficulties posed by asymptomatic malaria in pregnant women within the framework of the country's attempts to eliminate the illness.

## Method

### Design and protocol registration

The main emphasis of this systematic review and meta-analysis was the prevalence of asymptomatic malaria and associated risk factors among pregnant women in Ethiopia. The registration number for this systematic review is [CRD42023411385] in PROSPERO. To avoid duplications, we thoroughly searched for comparable systematic reviews and meta-analyses that have been published in Ethiopia on this topic.

### Selection and eligibility criteria

#### Criteria for inclusion

For this systematic review and meta-analysis, all literature on the prevalence of asymptomatic malaria and associated factors among pregnant women in Ethiopia was gathered. The fundamental information on the sample size, diagnostic techniques, prevalence of asymptomatic malaria infection, and its determinants in pregnant mothers in different regions of Ethiopia, as well as their status, was included in each study's original research publication, which was published in English. The investigations that made up this review were carried out in Ethiopia in community-based settings and medical facilities. Only research done on pregnant women in Ethiopia was taken into consideration for the review. We used cross-sectional studies that were published as journal articles and found in an academic database in the English language. There was no restriction on the year of publication. The review did not include abstracts from non-human studies or conferences.

### Database and searching strategy

Electronic resources like Google Scholar, PubMed, Scopes, the Web of Science, the Cochrane Library, and African Journals Online were used to conduct a thorough search of studies, both published and unpublished. “prevalence, magnitude, proportion, epidemiology, asymptomatic malaria, asymptomatic *Plasmodium* falciparum, asymptomatic *Plasmodium* vivax, associated factors, determinants, predictors, pregnant women, Ethiopia.” The investigation took place between April 1 and April 30, 2023. The meta-analysis was reported using the PRISMA (Preferred Reporting Items for Systematic Reviews and Meta-Analyses) criterion (18).

### Outcome measurement

The study's two main findings are as follows: A national assessment of the prevalence of asymptomatic malaria among pregnant mothers was the first result. The prevalence and its standard error were created and calculated using the metan prevalence standard error command. A rapid diagnostic test (RDT) and/or microscopy were performed to confirm the existence of asymptomatic malaria. The factors associated with asymptomatic malaria were the study's second finding. The data for this outcome were taken from a Microsoft Excel file in a two-by-two table format. Using the data from the initial study, the log odds ratio (OR) for each factor was then determined. These studies considered the following factors: IRS service, ITN use, the occurrence of stagnant water, residence (rural vs. urban), and education (literate or illiterate).

### Data extraction and quality assessment

A standardized Excel data extraction checklist was used by the three data extractors (T.D., E.T., and G.M.) to extract the data. The combined search results from the databases were combined, and duplicate articles were initially removed using reference management software (Endnote version X20). Based on their titles and abstracts, the studies were then evaluated and rejected. The remaining articles' full texts were scrutinized for eligibility based on the previously defined inclusion and exclusion criteria. The list of data to be extracted for the first result includes the name of the author, the year of publication, the study area (where the study was conducted), the study design, the way the outcome was measured, the sample size, the test method, the overall sample size, and the number of asymptomatic malaria cases (prevalence of asymptomatic malaria in pregnant women). Data was gathered in the form of two-by-two tables for the second outcome, or the factors linked to asymptomatic malaria. Following that, the log OR and selog OR were computed using the findings of the initial investigations. Disagreements between the three independent reviewers were resolved by bringing in a fourth reviewer after discussions for possible consensus (S.S.). The Newcastle-Ottawa Scale for cross-sectional studies served as the basis for the quality assessment tool for the study that was included (19).

### Data analysis and synthesis

Each original study's key information was extracted using a format made for a Microsoft Excel spreadsheet. After that, the information was exported for examination in STATA Windows version 15. The prevalence and standard error of prevalence, which were calculated using STATA's “generate” command, served as the primary outcomes for each study. The second result of each trial also contained the logarithm and standard error of the OR. A forest plot was used to represent the prevalence of asymptomatic malaria and the factors that contribute to it in pregnant women. The inverse variance index was used to assess the research's heterogeneity (I^2^). I^2^ values of 0%, 25%, 50%, and 75% were evaluated as appropriately indicating no, minimal, moderate, and substantial levels of heterogeneity (20). To determine the pooled prevalence of asymptomatic malaria and rule out publication bias, a funnel plot and a random effects model were used. Additionally, Egger's statistical test was used to determine the statistical significance of publication bias.

## Results

### Study selection and characteristics

**Figure 1:**
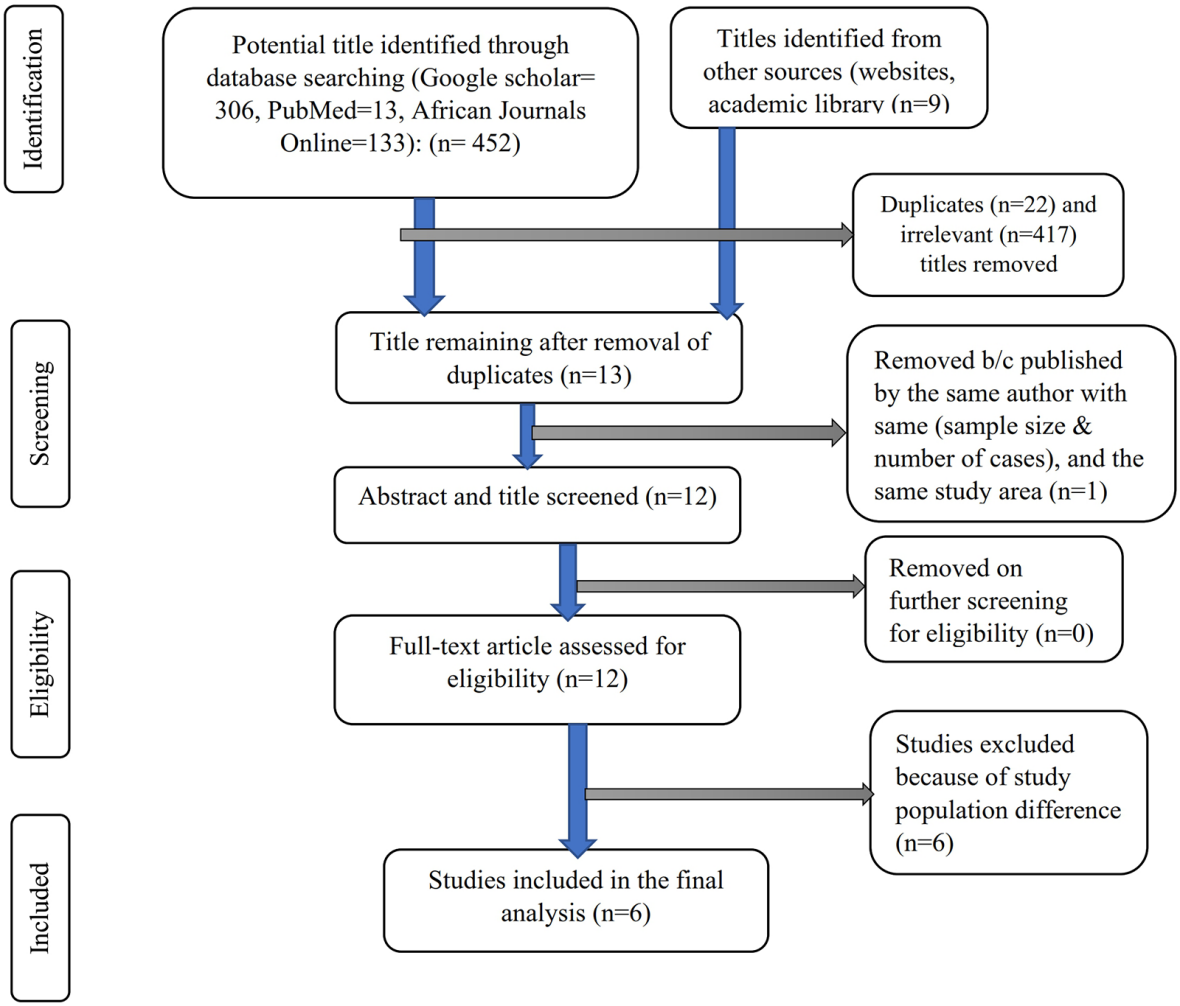
PRISMA flow diagram to present the study selection process.

From various electronic databases, 432 published papers were identified. Of the identified studies, 417 were removed due to irrelevant titles or not being conducted in Ethiopia, and 22 articles were excluded due to duplication. On further selection of the abstract and titles, 13 Ethiopian articles were identified, of which two studies were conducted by the same author in the same study area with the same sample size and number of cases (of the two, one study with better information was included), and 12 full-text articles were screened for eligibility, of which six were excluded (conducted among a population other than pregnant women). The final analysis included the remaining six studies that met the eligibility criteria. The PRISMA flow diagram was used to show the study selection process (Figure 1).

### Features of the included studies

The six papers examined in this review were cross-sectional studies on pregnant women that were written for journal publications. The Gurage Zone in southern Ethiopia had the smallest sample size (230), and the Merti area in Oromia had the biggest sample size, totaling 1,627 women who participated in the tests (364) (21). In the end, six studies were taken into consideration, including two from the Amhara Region (22, 23), two from Southern Ethiopia (24, 25), two from Oromia (21, 26), and four community-based studies (21, 23, 25, 26). Five of the studies employed microscopy and RDT as the outcome measurement, while one study (24) relied solely on microscopy, and the other five studies used both microscopy and RDT (21–23, 25, 26). While one of the microscopy tests' findings did not identify the *Plasmodium* species, five of them did (Table 1).

**Table 1:** Characteristics of studies included in this meta-analysis.

Authors (citation)	Year of publication	Study setting	Study period	Study design	Total sample size	No of cases and prevalence by microscopy *n* (%)	No of cases and prevalence by RDT *n* (%)	Test method
Nega et al. (25)	2015	Southern Ethiopia	2013	Community-based cross-sectional	341	31 (9.1)	33 (9.7)	Microscopy & RDT
Subussa et al. (21)	2021	Oromia	2018	Community-based cross-sectional	364	13 (3.6)	13 (3.6)	Microscopy & RDT
Feleke et al. (23)	2020	North-Shoa, Ethiopia	2018–2019	Community-based cross-sectional	263	15 (5.7)	9 (3.4)	Microscopy & RDT
Solomon et al. (24)	2020	Southern Ethiopia	2019	Facility-based cross-sectional	230	35 (15.2)	N/A	Microscopy & RDT
Balcha et al. (26)	2023	Oromia	2022	Community-based cross-sectional	328	9 (3.0)	10 (3.0)	Microscopy & RDT
Tilahun et al. (22)	2020	Northwest Ethiopia	2019	*Health facility-based cross-sectional*	331	30 (9.1)	17 (5.1)	Microscopy & RDT

### Information on the study site and period

The distribution of malaria in the study areas was considered, and accordingly, four studies were carried out from February to August, the period of low malaria transmission (22, 24–26). There is one study in the major malaria transmission season (November to January) (23), and one study was conducted in both the minor and major transmission seasons (March to September) (21).

### Prevalence of asymptomatic malaria

**Figure 2:**
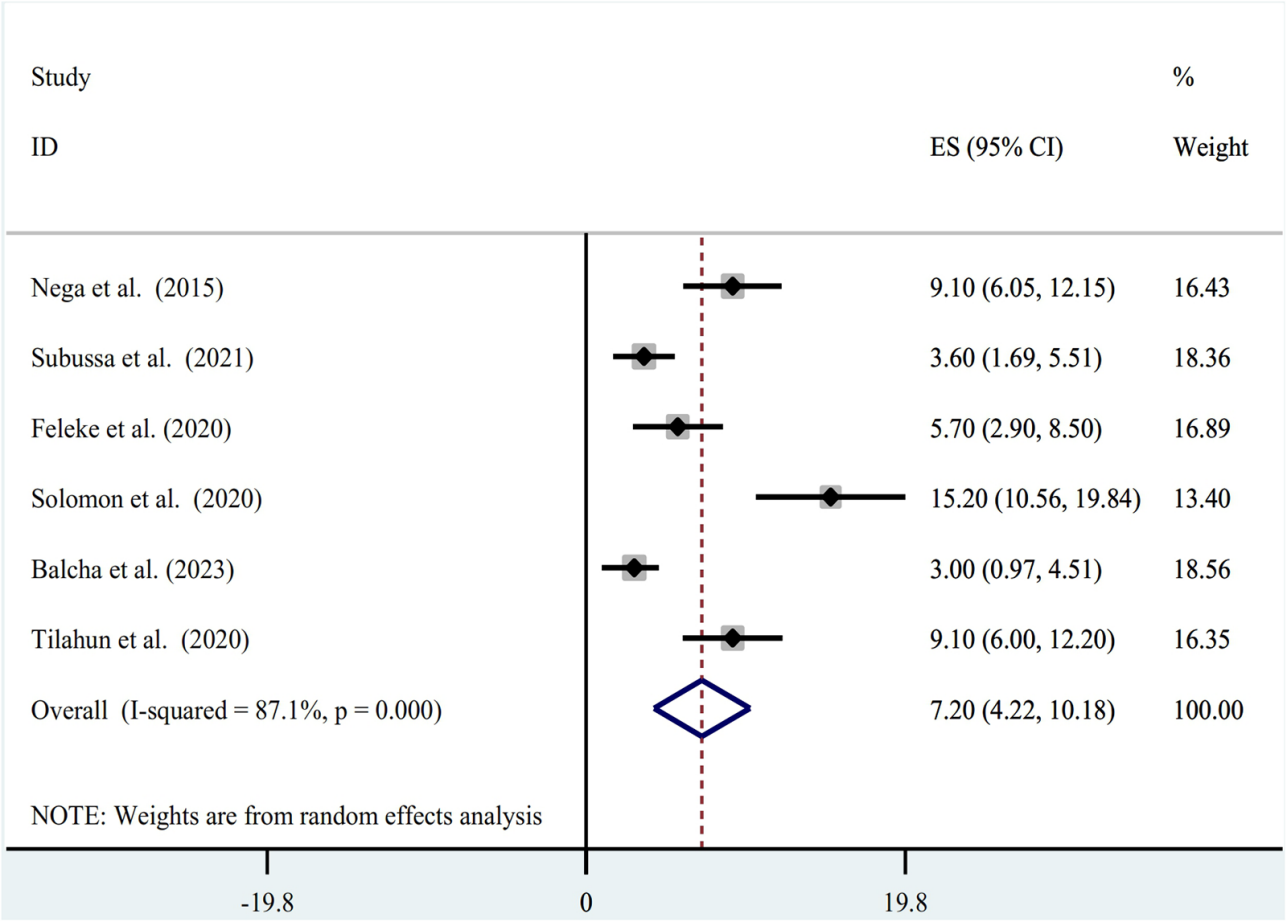
Forest plot of prevalence of asymptomatic malaria among pregnant women by microscopy in Ethiopia, systematic review and meta-analysis, 2023.

**Figure 3:**
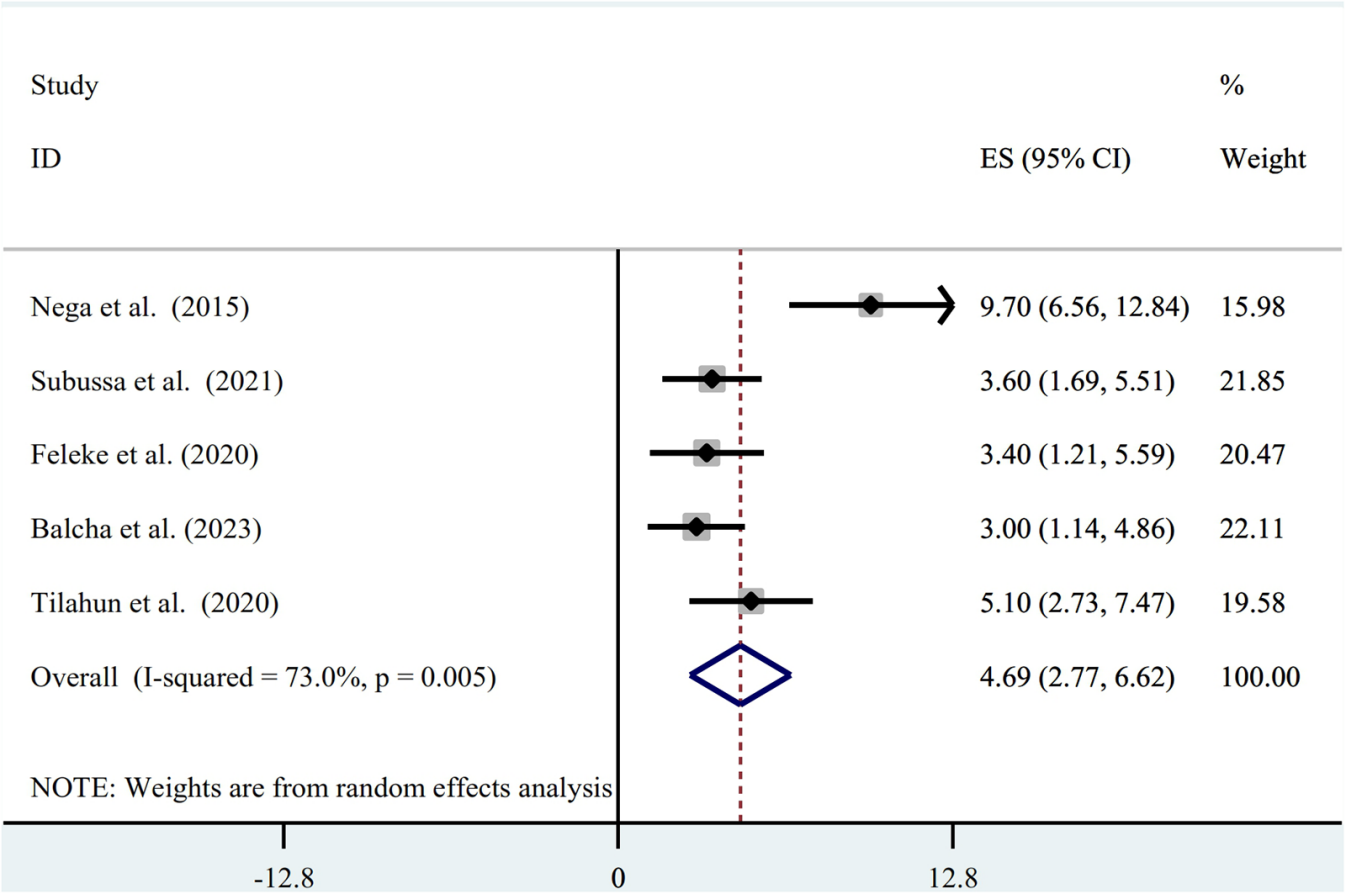
Forest plot of prevalence of asymptomatic malaria among pregnant women by RDT in Ethiopia, systematic review and meta-analysis, 2023.

**Figure 4:**
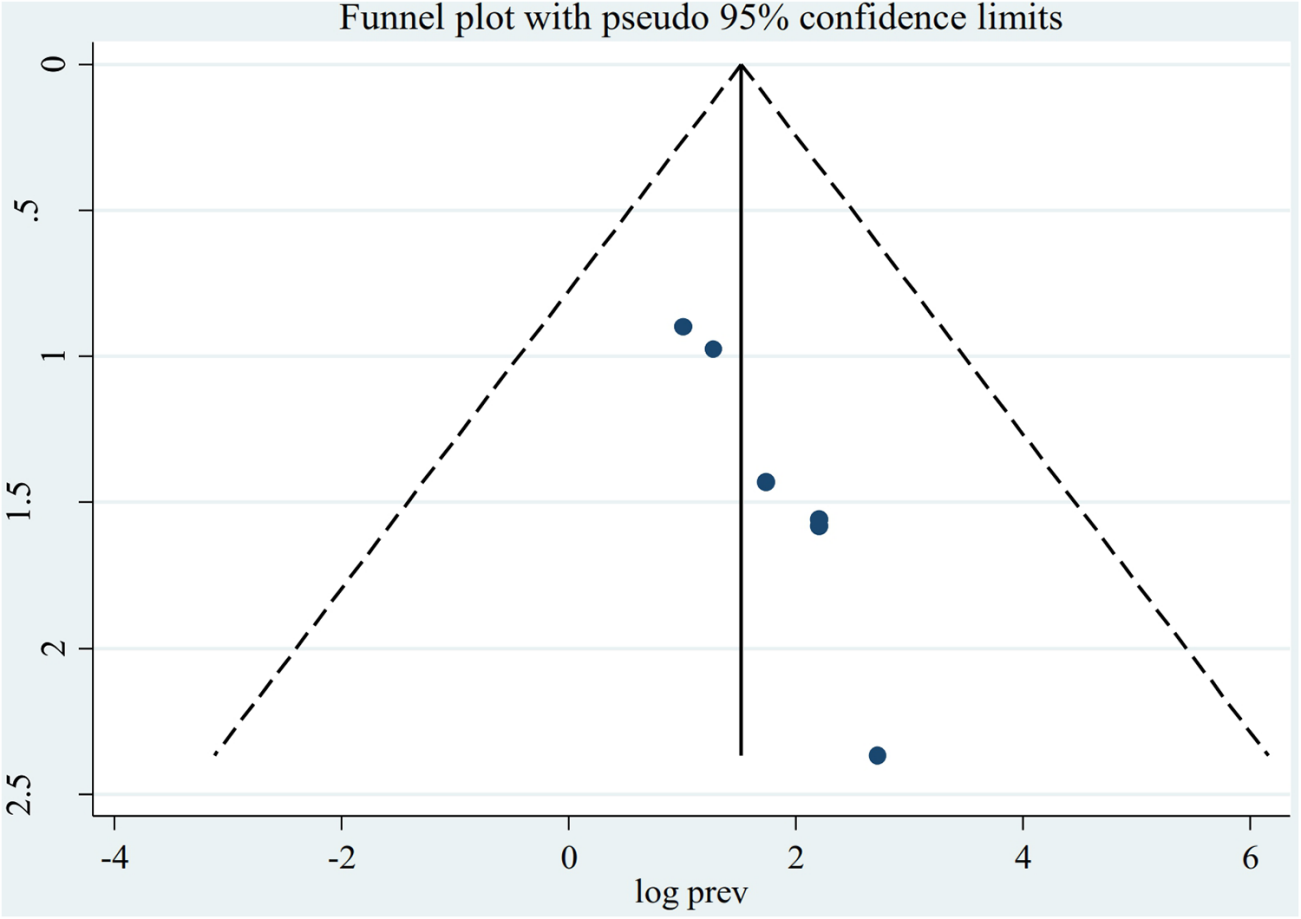
Funnel plot indicating the presence of publication bias, prevalence of asymptomatic malaria among pregnant women by microscopy in Ethiopia, systematic review and meta-analysis, 2023.

The random-effect model was used to determine the pooled prevalence of asymptomatic malaria among pregnant women, which was under study in various settings because we did not identify any significant heterogeneity between studies in our meta-analysis (*I*^2^ = 87.1%, *P*-value 0.000). The findings of the original study indicate that asymptomatic malaria prevalence among pregnant women in Ethiopia is variable and uncertain. The Gurage zone in southern Ethiopia had the highest prevalence (15.2%, 95% CI: 10.56, 19.84), and Oromia had the lowest (3.0%, 95% CI: 0.97, 4.51) (24) when applying the RDT results (Table 1). Asymptomatic malaria prevalence in pregnant women was 7.20 (95% CI: 4.22, 10.18) and 4.69 (95% CI: 2.77, 6.62), as determined by microscopy and RDT, respectively (Figures 2, 3). A graphical funnel plot and an Egger's test with a 5% level of significance were used to check for publication bias (Figure 4). The asymmetric funnel plot indicates no publishing bias. Additionally, the results of Egger's test (*P*-value = 0.053) indicated that there was no statistically significant publication bias (Table 2).

**Table 2:** Egger's test for publication bias.

Egger's test					
Std_Eff	Coef.	Std. err.	*t*	*P* > *t*	[95% Confidence interval]
Slope	−3.510976	1.27	−2.77	0.053	−7.024076,.0021234
Bias	5.933424	.8116859	7.31	0.002	3.679823, 8.187026

The results of the studies were pooled using a random-effects model, and sensitivity analyses were carried out to determine how the findings of one study would influence those of the others. There was no strong evidence to support the claim that one study had an impact on the other studies (Table 3).

**Table 3:** Asymptomatic malaria prevalence among pregnant women in Ethiopia determined by microscopy and RDT: sensitivity analysis, systematic review, and meta-analysis, 2023.

Study omitted	Study omitted estimate	[95% Conf. interval]
Microscopy		
Nega et al. (25)	6.83	3.56,10.10
Subussa et al. (21)	8.10	4.37, 11.82
Feleke et al. (23)	7.59	3.97, 11.21
Solomon et al. (24)	5.87	3.40, 8.34
Balcha et al. (26)	8.19	4.73, 11.65
Tilahun et al. (22)	6.83	3.57, 10.10
Combined	**7**.**20**	**4.22, 10.20**
RDT		
Nega et al. (25)	3.65	2.63, 4.68
Subussa et al. (21)	4.46	3.33, 5.60
Feleke et al. (23)	4.45	3.37, 5.54
Balcha et al. (26)	4.71	3.56, 5.86
Tilahun et al. (22)	4.06	2.99, 5.13
Combined	**4**.**24**	**3.26, 5.21**

The bold value 7.20 stands for the pooled prevalence of asymptomatic malaria among pregnant women in Ethiopia, whereas 4.24 is the combined or pooled prevalence of the disease as found by rapid diagnostic tests.

### Asymptomatic malaria and the factors associated among pregnant women in Ethiopia

#### Asymptomatic malaria and ITN utilization

**Figure 5:**
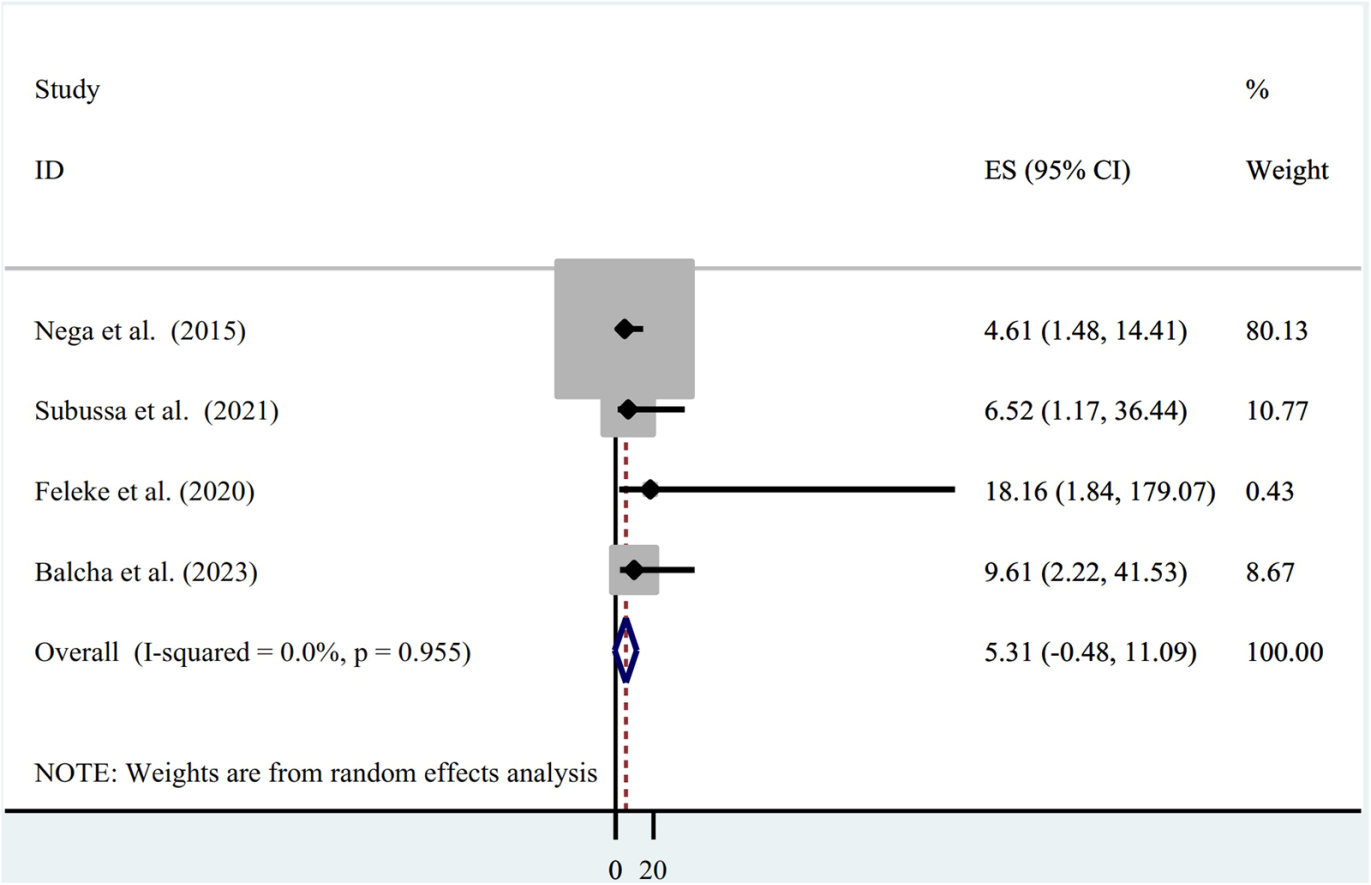
Association between asymptomatic malaria among pregnant women and insecticide-treated net utilization in Ethiopia, systematic review and meta-analysis, 2023.

The use of ITN and asymptomatic malaria infection were found to be associated in four of the studies (21, 23, 25, 26), of which all four studies identified showed a significant association (OR = 4.61; 95% CI: 1.48, 14.41), (OR = 6.52; 95% CI: 1.17, 36.44), (OR = 18.16; 95% CI: 1.84, 179.07), and (OR = 9.61; 95% CI: 2.22, 41.53). However, the pooled results did not show a significant association between asymptomatic malaria infection and ITN utilization (OR=5.31; 95% CI = −0.48, 11.09) (Figure 5).

#### Asymptomatic malaria and presence of stagnant water

**Figure 6:**
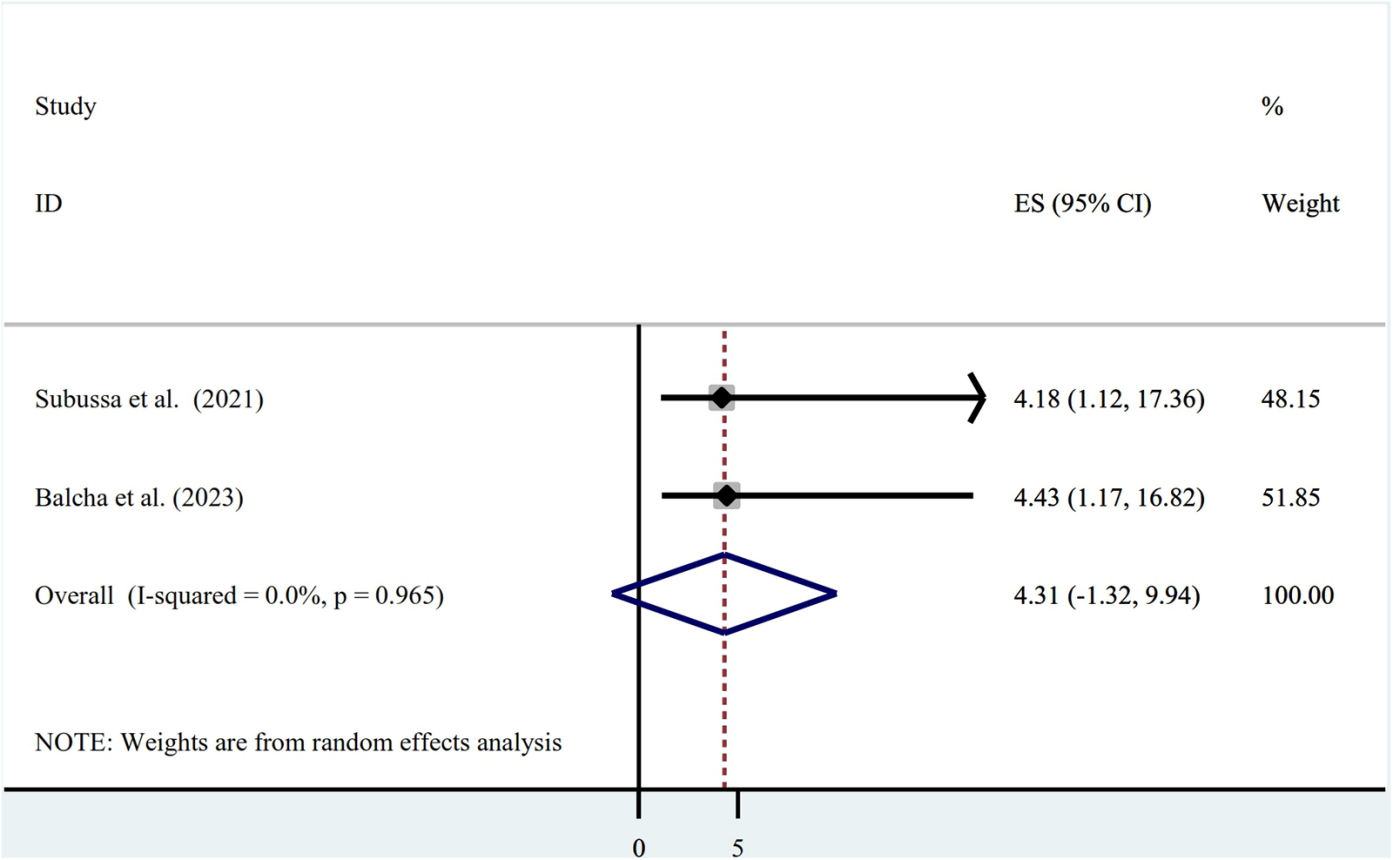
Association between asymptomatic malaria and presence of stagnant water among pregnant women in Ethiopia, systematic review and meta-analysis, 2023.

The presence of stagnant water and an asymptomatic malaria infection showed a statistically significant association in two studies that were included in the final analysis, with odds ratios of (OR = 4.18; 95% CI: 1.12, 17.36) and (OR = 4.43; 95% CI: 1.17, 16.82), respectively. The presence of stagnant water and asymptomatic malaria did not have a statistically significant correlation, according to the pooled estimate of the odds ratio (OR = 4.31; 95% CI: −1.32, 9.94). The presence of heterogeneity was investigated using a random-effects model (I2 = 0.0%, *p*-value = 0.965) (Figure 6).

#### Asymptomatic malaria and (IRS service, education level, and residence)

In this review, the authors tried to investigate the pooled estimate of associated factors such as IRS service, education level, and residence. To assess the association between IRS service and asymptomatic malaria, five full-text articles were chosen (21–23, 25, 26), of which four of the studies (21, 23, 25, 26) revealed no statistically significant relationship, while only one study (22) indicated a statistically significant relationship. Out of the six investigations, only one (26) discovered a statistically significant odds ratio for an association between educational status and asymptomatic malaria in pregnant women. As a result, the odds ratio was not pooled. The place of residence was also thought to be a potential contributing factor, but there are only two studies that provide comprehensive data on the prevalence of asymptomatic malaria among pregnant women in Ethiopia, and of those studies, only one (22) demonstrated a significant association with asymptomatic malaria. Hence, we are unable to compute the odds ratios' pooled estimate.

## Discussion

Malaria remains one of the primary public health issues in Ethiopia, despite the country's declining trend of prevalence (27). The global effort to eliminate malaria at the appropriate time, as well as in Ethiopia, is being greatly influenced and challenged by the rising trends of the disease's asymptomatic nature (14, 16, 28, 29). The first of its type in Ethiopia, this systematic review and meta-analysis examined asymptomatic malaria in pregnant women (vulnerable populations). In comparison to many relevant studies carried out in developed countries, there are comparatively few studies that are available and eligible for review regarding asymptomatic malaria in pregnant women in Ethiopia. The main objective of this analysis was to find out the pooled prevalence of asymptomatic malaria in pregnant women in Ethiopia and its contributing factors. To eliminate malaria in both low- and high-transmission settings, intervention that targets the parasite reservoir may be crucial. Asymptomatic malaria infection poses a quiet threat to the population and is a major factor in the disease's transmission (8).

Microscopy and RDT results revealed that the pooled result of asymptomatic malaria in pregnant women was 7.20 (95% CI: 4.22, 10.18) and 4.69 (95% CI: 2.77, 6.62), respectively. When compared to the 10.8% global asymptomatic malaria prevalence among pregnant women, the findings were much lower (10). The current finding is much lower compared to the one found in Sub-Saharan Africa and Nigeria, with a reported pooled prevalence of asymptomatic malaria of 26.1% (30) and 34.3% (31) respectively. The discrepancy could be attributed to the different sample sizes, diagnostic methods, and the fact that four of the six studies examined were carried out during Ethiopia's low-transmission season for malaria. A rapid diagnostic test was employed in other studies as a diagnostic technique that might raise the prevalence of (32). Additionally, the overall prevalence of the current findings was lower than that of pocket studies already out in the African countries of Ethiopia and Burkina Faso (33, 34).

Malaria morbidity has significantly decreased as a result of the enormous expenditure on antimalarial preventative treatments and advancements in diagnostic capability. The health extension program, which is consistent with the evidence as well (14, 16), strengthened the development of the health sector as a whole and, in particular, the primary health care unit.

Most importantly, given the Ethiopian government's determination to carry out a malaria elimination plan that could lead to a significant decrease in malaria prevalence through several strategies, including providing material and technical support to all regions of the country, coordinating regional capacity-building efforts in terms of manpower, logistics, and finances, and strong monitoring and evaluation (35), it is difficult to compare this analysis to research done at the global or African levels. There is no guarantee that the results from different parasite prevalence surveys will be comparable because they are frequently opportunistic, subject to observer bias, non-standardized, and may be influenced by a variety of factors like the timing of sampling with regional malaria transmission seasons and the methodology used to detect parasites (14, 35). Unlike other empirical studies, the authors of this review chose to carefully assess the decreased pooled prevalence in this case. Small-scale studies have revealed a prevalence of asymptomatic malaria in pregnant women in Ethiopia ranging from 3 to 15.2%, and it is impossible to completely rule out the possibility of other causes. Again, we were hedging against the likelihood that the pooled prevalence reported in our study would fairly represent the burden of malaria in real-world settings. This may be because more cases of asymptomatic malaria may exist than those identified with RDT and microscopy, given the availability of other cutting-edge diagnostic methods. Additionally, numerous other researchers used various definitions of asymptomatic malaria similar to those used in this review (35, 36).

The educational status of pregnant women was not found to be significantly associated with asymptomatic malaria in Ethiopia in this comprehensive review and meta-analysis. This result did not agree with those of previous investigations (11, 33, 37). The variations can be attributable to the study design (study area, sample size, and diagnostic methods), as well as the sample's timing relative to the local malaria transmission seasons (14). Given that temperature directly affects malaria transmission in Ethiopia, it is distributed variably based on altitude and experiences biannual peaks in temperature (38). Besides, the authors were unable to find studies that compared and contrasted the identified associated factors using a thorough methodology. This is a result of the methods utilized as a preventive strategy by building physical barriers, deterring or eliminating mosquitoes, and killing any that may survive without requiring additional chemical treatments (38).

## Limitations

The Newcastle-Ottawa Scale for Cross-Sectional Studies was used to assess the quality of each study after a thorough search of the literature for published and unpublished studies. This will make it easier for the review to come to relevant conclusions. For the country to eliminate malaria by 2030, healthcare policymakers in the nation must carefully consider the results of this study. One should use caution when interpreting the results, nevertheless, due to some of this review's shortcomings. The studies' diagnostic approaches—microscopy and RDT have limitations in their ability to identify all asymptomatic malaria cases. This could lead to an inaccurate generalization of the decreased pooled prevalence observed in this study.

## Conclusion

This study's findings highlighted a high pooled prevalence of asymptomatic malaria. People who did not use ITNs and those who lived close to stagnant water had six- and four-times higher rates of asymptomatic malaria, respectively. We recommend better environmental management and ITN use. The prevalence of the disease may be higher if more accurate diagnostic techniques are used. A study that considers variables throughout the actual causal chain is required to uncover more useful components. To effectively intervene in the elimination of malaria, proactive case detection is also encouraged.

## Data Availability

The original contributions presented in the study are included in the article/Supplementary Material, further inquiries can be directed to the corresponding author.
